# Tear proteome analysis in ocular surface diseases using label-free LC-MS/MS and multiplexed-microarray biomarker validation

**DOI:** 10.1038/s41598-017-17536-2

**Published:** 2017-12-12

**Authors:** Javier Soria, Arantxa Acera, Jesús Merayo-LLoves, Juan A. Durán, Nerea González, Sandra Rodriguez, Nikitas Bistolas, Soeren Schumacher, Frank F. Bier, Harald Peter, Walter Stöcklein, Tatiana Suárez

**Affiliations:** 1 Bioftalmik Applied Research, Bizkaia Science and Technology Park, Building 612, E-48160 Derio, Bizkaia Spain; 2 Instituto Universitario Fernández-Vega, Avda Dres Fernández-Vega num 34, Oviedo, E-33012 Principado de Asturias Spain; 3 Instituto Clínico Quirúrgico de Oftalmología (ICQO), Virgen de Begoña N° 34, E-48006 Bilbao, Bizkaia Spain; 4 Department of Ophthalmology, School of Medicine, University of the Basque Country (UPV/EHU), Leioa, Bizkaia Spain; 5 Department of automatization, Fraunhofer Institute for Cell Therapy and Immunology, Branch Bioanalytics and Bioprocesses, Am Muehlenberg 13, 14476 Potsdam-Golm, Germany

## Abstract

We analyzed the tear film proteome of patients with dry eye (DE), meibomian gland dysfunction (MGD), and normal volunteers (CT). Tear samples were collected from 70 individuals. Of these, 37 samples were analyzed using spectral-counting-based LC-MS/MS label-free quantitation, and 33 samples were evaluated in the validation of candidate biomarkers employing customized antibody microarray assays. Comparative analysis of tear protein profiles revealed differences in the expression levels of 26 proteins, including protein S100A6, annexin A1, cystatin-S, thioredoxin, phospholipase A2, antileukoproteinase, and lactoperoxidase. Antibody microarray validation of CST4, S100A6, and MMP9 confirmed the accuracy of previously reported ELISA assays, with an area under ROC curve (AUC) of 87.5%. Clinical endpoint analysis showed a good correlation between biomarker concentrations and clinical parameters. In conclusion, different sets of proteins differentiate between the groups. Apolipoprotein D, S100A6, S100A8, and ceruloplasmin discriminate best between the DE and CT groups. The differences between antileukoproteinase, phospholipase A2, and lactoperoxidase levels allow the distinction between MGD and DE, and the changes in the levels of annexin A1, clusterin, and alpha-1-acid glycoprotein 1, between MGD and CT groups. The functional network analysis revealed the main biological processes that should be examined to identify new candidate biomarkers and therapeutic targets.

## Introduction

DE and MGD diseases involve the disruption of the lacrimal functional unit, resulting in symptoms of discomfort and visual disturbance and tear film instability. These illnesses may exist independently as either symptomatic or asymptomatic disorder; however, they are frequently found in the same patient. The progression of these diseases typically leads to alterations in the tear film and tear hyperosmolarity^1^ and the secretion of inflammatory mediators into the tears, initiated by cytokine release and metalloproteinase activation^2^. Ocular dryness disorders also promote squamous metaplasia (SM), mainly as a consequence of inflammation^3^, whose severity can be used for grading different diseases affecting the ocular surface^4,5^.

Tear film proteomics offers powerful analytical tools for studying the proteins involved in ocular diseases. Using these tools, we might be able to establish the precise functions of these proteins in the underlying pathophysiological processes and provide diagnostics biomarkers. The studies of differential protein expression in complex biofluids such as tear film require rapid, highly reproducible, and accurate quantification. These requirements can be satisfied by mass spectrometry-based analysis^6^. Label-based mass spectrometry techniques have been employed to analyze the tear film, mainly by using isobaric tags for relative and absolute quantitation (iTRAQ)^7–10^. However, label-free shotgun proteomics techniques are becoming increasingly popular since they are faster and supply cleaner and simpler results than the label-based techniques^6^. One of the most used label-free methods is spectral counting of identified proteins. This method is based on the fact that the number of spectra corresponding to peptides originating from a given protein shows a good linear correlation with the abundance of that protein^11–14^. Nevertheless, count ratios can be biased because of particular physiochemical properties of peptides or sampling issues in Data-Dependent Acquisition (DDA) shotgun procedure that may affect their detection by MS. As not all peptides can be perfectly ionized, appropriate measurement corrections should be applied to obtain accurate data^15,16^.

To date, several proteomic techniques have been applied to study variations in tear proteome caused by pathological conditions and/or medical treatments. Many MS-based proteomic studies of tear film have been carried out using iTRAQ^7–10^, label-free^14,17–19^, SELDI-TOF^20^, or MALDI-TOF^21–23^ analysis. However, the results have been, at times, conflicting. Some of the studies have demonstrated changes in the expression of several proteins. Overexpression has been reported for protein ANXA1^14,23–25^, S100A9^7,14,24,25^, S100A8^7,14,20,23–25^, and S100A4^7,24^ and downregulation, for prolactin-inducible protein (PIP)^7,8,24–26^, lactotransferrin (LT)^7,8,14,24,27^, proline-rich protein 4 (PRP4)^8,14,20,25,26^, lysozyme (LYZ)^7,8,14,20,25,27^, and cystatin-S (CST4)^8,14,24,25^, among others. Nevertheless, the changes in the expression of some proteins remain controversial. For example, several studies^20,27^ have demonstrated upregulation of lipocalin-1 (LCN1), but its downregulation has been shown in others^7,8,24,28^. Similarly, conflicting results have been reported for other proteins such as secretoglobin 2A2^8,26,28^ and serum albumin^14,20,26–28^. These discrepancies might have been caused by several factors such as different sample collection methods or sensitivity and dynamic range of the employed quantitation techniques^29,30^. Therefore, more extensive tear proteome studies focusing on systems biology, supported by orthogonal validation assays, are needed to extract meta-information common to all these studies. The results of such studies should reduce the technological and experimental bias and improve our knowledge of the role of relevant proteins in the pathophysiology of ocular surface-related disorders.

We have previously characterized tear film proteomes and identified biomarkers for the aqueous-deficient dry eye (ADDE) and MGD, the main cause of evaporative DE (EDE) condition^31^, using 2D gel-based proteomics^24^.

The purpose of the present study was to identify proteins that can be used for discrimination between DE and MGD pathologies. To achieve this, we performed a quantitative differential study of tear protein expression using LC-MS/MS spectral-counting quantitative proteomics. Three candidate biomarkers were validated employing an orthogonal technique (customized microarray assays) using independent DE and CT tear samples.

## Materials and Methods

### Patients

**Figure 1:**
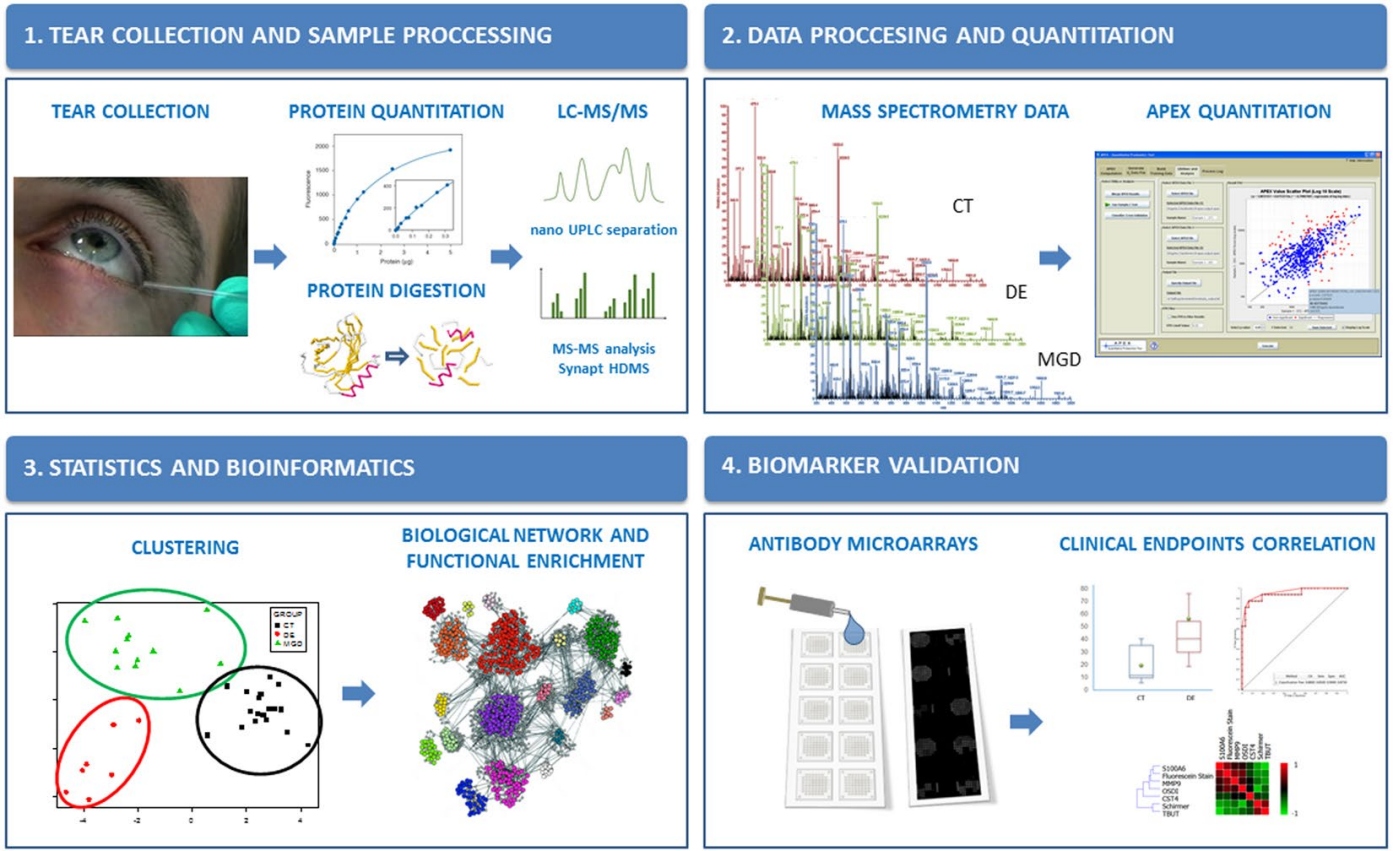
Workflow overview. A general workflow outlining the steps used in the study for the identification of proteins deregulated in DE and MGD tear samples, using LC-MS/MS label-free quantitative proteomics, candidate biomarkers validation, and clinical correlation strategy.

A retrospective case-controlled study was carried out, in which 70 patients were enrolled. The experimental design consisted of a discovery phase (including 7 DE patients, 12 chronic MGD patients, and 18 CT individuals for biomarker candidate selection), and a validation phase, for which 24 DE patients and 9 CT individuals were recruited (Fig. 1).

This research was conducted by medically qualified personnel after approval by the Cruces Hospital and the Principado de Asturias Hospital Ethics Committees. Approval was obtained in strict accordance with the tenets of the Declaration of Helsinki on Biomedical Research Involving Human Subjects. Patients were recruited at the Instituto Clínico Quirúrgico de Oftalmología (Bilbao, Bizkaia, Spain) and the Instituto Universitario Fernández-Vega (Oviedo, Asturias, Spain). Before tear collection, the signed informed consent was obtained from all patients, once the nature and possible consequences of the study had been explained. The diagnosis was based on clinical examination including the Schirmer I test with anesthesia to measure the basal secretion, slit-lamp examination of the lid margin and Meibomian glands, fluorescein staining results according to Oxford scale, and subjective symptoms. Each patient answered the OSDI questionnaire, which included some statements about the quality of their vision and wellbeing.

Patients were classified as having DE if they had dry eye symptoms, dynamics abnormalities in Schirmer I test (≤5 mm/5 min), characteristic fluorescein staining of the corneal epithelium, and a tear breakup time (TBUT) ≥5 s. MGD was diagnosed if the patients had eyelid inflammation, Schirmer I test results >5 mm/5 min, TBUT <5 s and alterations in Meibomian glands. Healthy subjects in the CT group were not suffering from any ocular disease (no allergic or atopic history). They presented Schirmer I test values >5 mm/5 min, TBUT >5 s, no corneal fluorescein staining or sensations of discomfort, and no evident eyelid inflammation. The individuals with (or history of) any systemic or ocular disorder or condition (including ocular surgery, trauma, and disease) and patients with Sjögren’s syndrome were excluded. Contact lens users were also excluded to avoid any possible interference with the interpretation of the results.

### Clinical evaluation of patients and sample collection

The order of tests and sample collection was always the same. First, the OSDI questionnaire was conducted to assess the symptoms of ocular irritation and their effect on the vision-related function. Second, one drop of topical anesthetic was applied to the ocular surface and the Schirmer I and fluorescein tests were performed. The tear samples and conjunctival impression cytology samples were collected one day later to avoid the interference between clinical tests and the proteomic study.

All tear samples were collected using calibrated 10-µl glass microcapillary tubes (BLAUBRAND intraMark, Wertheim, Germany). Tear samples were obtained from the inferior temporal tear meniscus, minimizing irritation of the ocular surface or lid margin, without anesthesia. After collection, the tear samples were placed in Eppendorf tubes and stored at −80 °C until analysis. Protein quantification was performed using the EZQ Protein Quantification Kit (Invitrogen Dynal AS, Oslo, Norway).

To evaluate the degree of damage of the conjunctival epithelium (by examining the extent of SM), conjunctival impression cytology (CIC) samples were obtained for PAS-hematoxylin staining. To achieve this, 5 × 5-mm membranes of cellulose acetate (HAWP304, Millipore, Bedford, MA, USA) were applied to the bulbar superior conjunctival epithelium following topical anesthesia (Colircusi double anesthetic, Alcon Cusí, Barcelona, Spain). The samples were immediately fixed in 96% ethanol and stained with PAS-hematoxylin, as previously described^32^. The samples were later examined under a light microscope to evaluate the grade of SM^33^. The cytoplasmic and nuclear areas of non-secretory cells, cytoplasmic alterations and staining, nuclear alterations, the nuclear to cytoplasmic area ratio (N:C ratio), and the number of goblet cells were examined.

### Tear protein digestion, LC-MS/MS, and protein identification

To digest the proteins, RapiGest SF (Waters, Milford, MA, USA) was added to 4 µg of total tear protein at a concentration of 0.25% (w/v), and the mixture was heated at 85 °C for 15 min with agitation. Proteins were reduced (5 mM dithiothreitol, 60 °C, 25 min), alkylated (15 mM iodoacetamide, room temperature, 30 min), and digested with trypsin (0.2 µg, 37 °C, overnight; Roche Diagnostics, Penzberg, Germany). Finally, RapiGest SF was inactivated following the manufacturer’s instructions.

LC-MS/MS analysis was performed in a nanoACQUITY UPLC system interfaced to a SYNAPT HDMS mass spectrometer (Waters Corporation, Milford, MA). An aliquot of 1 µg of protein from each sample was loaded onto a Symmetry 300 C18, 180 µm × 20 mm precolumn (Waters Corporation, Milford, MA, USA) and washed with 0.1% formic acid for 3 min at a flow rate of 5 µl/min. The precolumn was connected to a BEH130 C18, 75 µm × 200 mm, 1.7 µm (Waters Corporation, Milford, MA, USA), equilibrated in 3% acetonitrile with 0.1% formic acid. Peptides were directly eluted with a 120-min linear gradient of 3–60% acetonitrile onto a homemade nanoESI emitter. Data-dependent MS/MS acquisitions were performed on the 3 most intense precursors per scan, with charge states of 2, 3, or 4 over a survey m/z range of 400–1990 and a dynamic peak exclusion of 30 s. Collision energies were varied as a function of the m/z and charge state of each peptide. [Glu1]-fibrinopeptide B (Sigma-Aldrich, St. Louis, MO, USA) at a concentration of 100 fmol/ml was sprayed through the NanoLockSpray source and sampled every 30 s. The obtained spectra were processed using VEMS^34^ and searched using MASCOT version 2.2.03 (Matrix Science, London, UK) against the UniProtKB/Swiss-Prot (version 2015_12) database, using only Homo sapiens entries. For protein identification, the following parameters were adopted: carbamidomethylation of cysteines as fixed modification, oxidation of methionines as variable modification, peptide mass tolerance of 50 ppm, fragment mass tolerance of 0.1 Da, and 1 missed cleavage.

### Quantitative protein analysis using spectral counting (APEX quantitation)

Protein quantitation was performed using label-free spectral counting, employing APEX Quantitative Proteomics Tool v.1.1 as described previously^35^. MASCOT output files were converted to the pepXML format using Trans-Proteomic Pipeline software v. 3.2.2 (Institute for Systems Biology, Seattle, WA)^36^. The resulting pepXML files were analyzed using PeptideProphet^37^. Subsequently, ProteinProphet^38^ was employed to assemble peptides into protein identification groups. Only proteins with ProteinProphet probability ≥ 0.95 and at least two identified peptides with a PeptideProphet probability ≥ 0.95 were reported. A probability-based penalty for peptide detection was applied, given that some peptides are detected by MS more readily than others. APEX quantitation analysis involves three steps. In step 1, a classification model is built, based on physiochemical properties of the peptide sequence (peptide mass, length, amino acid composition, properties related to charge, hydrophobicity, and amino acid frequencies within the secondary peptide structures). The model is used to estimate MS-detectability (O(i)) for any given protein. Step 2 uses spectral count information to obtain absolute abundances of proteins in each MS/MS experiment, corrected by applying an MS-detectability correction factor. Step 3 allows statistical significance analysis of differential expression in distinct biological samples. The protein false discovery rate was set at 1%; only proteins identified at a 99% confidence level were used for spectral counting.

### Statistics

Data matrix obtained from APEX quantitation was used for multivariate statistical analysis. Before statistical analysis, protein expression data were filtered by considering only the proteins identified in at least 50% of biological samples. K-nearest neighbor data imputation was performed to complete the data matrix and manage missing values imposed by intrinsic sampling issues in DDA shotgun approaches. Data were then normalized using total spectral count normalization to reduce run-to-run variation^39^. Once the data matrix had been prepared for analysis, feature subset subtraction was performed by stepwise discriminant analysis with a p-value cutoff of 0.01, to obtain the most significant attributes defining each group. This process extracts the information that contributes most to the discrimination between groups. To reduce the dimensionality of the dataset for 2D-visualization including the possible overlap between the groups, sample clustering was performed (using an exploratory data technique, the canonical discriminant analysis). Normalization, imputation, feature subset, and clustering analysis were carried out using R-Package v2.15.2^40^.

### Functional network analysis

Once the most significant tear proteins had been identified by LC-MS/MS analysis, the list of the most significantly upregulated or downregulated proteins was loaded into Cytoscape v. 2.8.3^41^ using Reactome Functional Interaction Network Cytoscape plugin^42^, to find network patterns related to diseases. The Cytoscape plugin accesses the Reactome Functional Interaction Network database. This database extends curated pathways with non-curated sources of information, including protein–protein interactions, gene coexpression, protein domain interaction, Gene Ontology (GO) annotations, and text-mined protein interactions. The process obtains a biological network based on the experimentally found proteins and the directly related proteins required to connect them in a network. With this information, functional interaction sub-networks (modules) were constructed. Then, the analysis of network modules of highly interacting groups of proteins and the functional enrichment and pathway analysis were performed. To select the biological processes and/or pathways that are predominant in each of the modules, we filtered the functional enrichment results using a false discovery rate cutoff value of 0.01. Finally, the Hubba Cytoscape Plugin^43^ was applied to the functional interaction subnetwork to find the most important nodes/hubs within the network. Topological analyses were performed using the Edge Percolated Component ranking method to evaluate node essentiality.

### Validation of candidate biomarkers using antibody microarray assays

Three proteins with significant associations in mass spectrometry and network analysis (S100A6, CST4, and MMP9) were evaluated using customized sandwich-like antibody microarrays. The process of customized microarray preparation for quantitative tear biomarker analysis included several steps. These were i) identification of compatible antibody pairs for each biomarker and selection of high-affinity pairs by surface plasmon resonance (SPR) and sandwich ELISA assays, ii) evaluation of cross-reactivity between components in the multiplexed immunoassay, iii) determination of detection limits for each biomarker, iv) integration into the microarray platform and technical validation, and v) measurement of biomarker concentration in the selected tear samples (for validation purposes).

Briefly, mouse monoclonal antibodies against the selected proteins were spotted on 3D-epoxy-activated glass slides and cyclo-olefin polymer slides (PolyAn GmbH, Berlin, Germany) with the non-contact spotter sciFLEXARRAYER S11 (Scienion AG, Berlin, Germany), in a 12-subarray format. Each antibody was spotted 20 times to reduce the technical variability. The nozzle used was PDC90, and a voltage of 82 V was applied, with a pulse width of 46 µs, loading a final volume of 1.5 nl/spot. The arrays were blocked with TBS-T supplemented with BSA (1%) for 1 hour. Antigens S100A6, CST4, and MMP9 were incubated for 1 h to obtain a standard curve from 0.195 to 166 ng/ml. The reaction volume was 70 µl/well for all the steps in the immunoassay. Tear samples were diluted (1/30) in PBS for microarray analysis. Subsequently, the samples were incubated for 1 h with rabbit detection antibodies. Finally, after washing the slides with TBS-T, the secondary Alexa Fluor 647-labeled anti-rabbit antibodies were added and incubated for 1 h. Fluorescence of the spots was measured using a Tecan LS Reloaded Microarray Scanner (Tecan Deutschland GmbH, Crailsheim, Germany) at 633 nm, and protein concentration was determined based on standard curve intensity values.

After testing for normal distribution, significant differences between the groups were determined using Kruskal–Wallis non-parametric test. Once the concentration of each protein had been obtained, ROC curve analysis was performed, using logistic regression. The classification algorithm was trained using the biomarker panel, and to assess learner performance, a 10-fold random sampling was carried out with 70% of the samples as a training set. The remaining 30% of the samples constituted a test set. Statistical analysis was carried out using the Orange Canvas statistics package, version 2.6 (http://orange.biolab.si/). Finally, the Pearson correlation analysis was performed to assess correlations between clinical parameters and protein levels. The statistical analysis was conducted using R statistical program^40^.

### Data availability

The datasets generated during and/or analyzed during the current study are available from the corresponding author on reasonable request.

## Results

### Patients

The patients recruited for the study were grouped into two sub-study groups, for discovery and validation. In the first group (37 volunteers), tear proteomics and CIC analyses were conducted. Demographical and clinical data for the discovery phase are presented in Table 1.

**Table 1:** Demographic and clinical data of patients included in the discovery phase of the study.

Group	n	Gender (M/F)	Age	Schirmer I test	Squamous metaplasia grade				
					0	1	2	3	4
CT	18	8/10	44.6 ± 21.4	13.8 ± 3.5	13	4	1	0	0
MGD	12	5/7	54.7 ± 11.6	8.0 ± 2.2	2	4	2	2	2
DE	7	2/5	56.1 ± 14.8	1.7 ± 2.0	0	1	3	2	1

CT = Control; MGD = Meibomian gland dysfunction; DE = Dry eye.

We followed the recommendations for tests appropriate for diagnosis and assessment of the target ocular surface diseases studied here, DE and MGD^44^. Various diagnostic tests were used, depending on the objective. Thus, in the first part of the study (biomarker candidate selection), we analyzed patients with DE and MGD. We used the symptoms as a screening tool (OSDI), tear volume assessment to ascertain the subtype classification of DE (Schirmer test), and damage to the ocular surface (impression cytology) as a standard test to study squamous metaplasia and goblet cell density of the conjunctiva. In the second part of the study (the validation of candidate biomarkers), we assessed the DE patients and CT subjects; we employed the recommended diagnostic tests for DE^44^. Following the clinical protocol proposed for DE diagnosis, a broad range of diagnostic tests was performed, including OSDI (ocular surface symptoms), TBUT (tear film stability), Schirmer I test (tear volume), and fluorescein staining (damage to the ocular surface). This was also done to determine the correlation matrix for biomarker concentrations and clinical parameters.

PAS-hematoxylin impression cytology revealed that in control samples, 72% of individuals (13/18) presented grade 0 SM (G0), whereas the remaining 28% (5/18) showed grades 1–2 (Table 1). In the MGD group, 17% of patients (2/12) had SM of grade 0, whereas mild grades (G1 and G2) were seen in 50% (6/12), and moderate (G3) to severe (G4) grades in 33% of patients (4/12). In the DE group, a mild grade of SM was found in 57% of patients (4/7), while the remaining 43% of DE patients exhibited moderate (G3) and severe (G4) grades. Schirmer test results showed significant differences (p-value < 0.05) between the groups, with values of 1.7, 8.0, and 13.8 for the DE, MGD, and CT groups, respectively. Mean age differences between the groups were not statistically significant (p-value > 0.05).

In the second part of the study, tear samples from 33 patients were analyzed on microarrays for validation purposes. To evaluate the correlation between biomarkers and clinical parameters, OSDI, TBUT, Schirmer I test, and fluorescein staining results were obtained for each patient in this phase of the study. All these parameters differed significantly between DE and CT individuals. The mean values for each parameter are presented in Table 2.

**Table 2:** Demographic and clinical data of patients included in the validation phase of the study.

Group	n	Gender (M/F)	Age	OSDI	TBUT	Schirmer I test	Fluorescein staining
CT	9	3/6	36.78 ± 20.41	2.39 ± 2.45	13.39 ± 5.97	18.06 ± 5.85	0.33 ± 0.50
DE	24	10/14	54.58 ± 21.55	35.92 ± 19.37	6.21 ± 3.51	5.58 ± 5.30	1.50 ± 1.25

CT = Control; DE = Dry eye.

### Quantitative protein analysis using spectral counting

Quantitative LC-MS/MS spectra analysis of tear proteome was performed computationally using the adjusted spectral counting method.

The analysis identified 603 significantly distinct proteins, by filtering the data to a minimum of two peptides identified with a PeptideProphet probability of 0.9 and a ProteinProphet probability of 0.95. However, as only the proteins identified in at least 50% of the samples were considered, this number was reduced to 135 proteins.

Protein expression data for those 135 proteins were used in further analysis. Model-based data imputation was performed to complete the data matrix and manage missing values. To establish which proteins were characteristic for each pathological condition, a subtraction of expression data, using stepwise discriminant analysis modeling, was performed. As a result, we obtained a list of 26 specific proteins that undergo expression changes, achieving an effective discrimination between the groups (Table 3).

**Table 3:** Proteins identified by LC-MS/MS with the most significant changes in their expression levels in dry eye (DE), Meibomian gland dysfunction (MGD), and control (CT) groups.

UniProt	Protein name	Gene name	FDR (q-value)	FOLD	Highest mean	Lowest mean	Number of peptides
P01024	Complement C3	C3	5.66E-05	1.86	DE	CT	6
P06703	Protein S100-A6	S100A6	5.40E-08	2.26	DE	CT	2
P05109	Protein S100-A8	S100A8	1.68E-04	2.15	DE	CT	4
P00450	Ceruloplasmin	CP	3.76E-02	3.25	DE	CT	2
P05090	Apolipoprotein D	APOD	8.68E-03	3.85	DE	CT	3
P19652	Alpha-1-acid glycoprotein 2	ORM2	1.69E-04	2.13	DE	CT	4
P10599	Thioredoxin	TXN	4.14E-04	1.61	DE	MGD	3
P01857	Ig gamma-1 chain C region	IGHG1	4.14E-04	1.61	DE	MGD	10
P14555	Phospholipase A2, membrane-associated	PLA2G2A	1.77E-02	3.13	DE	MGD	7
P01009	Alpha-1-antitrypsin	SERPINA1	4.05E-03	1.98	DE	MGD	5
P03973	Antileukoproteinase	SLPI	2.32E-02	3.59	DE	MGD	3
P04083	ANXA1 protein	ANXA1	1.46E-03	1.83	MGD	CT	2
P10909	Clusterin	CLU	1.52E-02	2.68	MGD	CT	8
P02763	Alpha-1-acid glycoprotein 1	ORM1	1.75E-02	1.49	MGD	CT	4
P22079	Lactoperoxidase	LPO	1.73E-02	2.40	MGD	DE	2
Q99935	Proline-rich protein 1	PROL1	5.82E-03	1.64	CT	DE	8
O95968	Secretoglobin family 1D member 1	SCGB1D1	2.00E-02	1.63	CT	DE	5
O75556	Mammaglobin-B	SCGB2A1	3.56E-03	1.65	CT	DE	11
P31025	Lipocalin-1	LCN1	3.61E-02	1.81	CT	DE	11
P01036	Cystatin-S	CST4	3.56E-03	1.65	CT	DE	6
P12273	Prolactin-inducible protein	PIP	3.69E-02	1.9	CT	DE	8
P81605	Dermcidin	DCD	6.27E-03	2.55	CT	DE	2
P02814	Submaxillary gland androgen-regulated protein	PROL3	3.44E-02	1.61	CT	DE	4
Q08380	Galectin-3-binding protein	LGALS3BP	4.61E-02	2.64	CT	DE	2
Q9GZZ8	Extracellular glycoprotein lacritin	LACRT	2.19E-02	1.83	CT	DE	6
Q16378	Proline-rich protein 4	PRR4	3.23E-02	2.02	CT	DE	4

The fold-values shown for each protein refer to the ratios between the group with the highest mean expression values (Highest Mean) and the group with the lowest mean expression values (Lowest Mean). The number of peptides identified was filtered using the PeptideProphet probability threshold of 0.95. FDR = False discovery rate.

The clearest discrimination between the groups could be obtained by examining the expression of the following proteins (overexpressed in the DE group): complement C3 (C3), protein S100-A6 (S100A6), protein S100-A8 (S100A8), ceruloplasmin (CP), apolipoprotein D (APOD), alpha-1-acid glycoprotein 2 (ORM2), thioredoxin (TXN), Ig gamma-1 (IGHG1), membrane-associated phospholipase A2, (PLA2G2A), alpha-1-antitrypsin (SERPINA1), and antileukoproteinase (SLPI). Of these proteins, TXN, IGHG1, PLA2G2A, SERPINA1, and SLPI showed the lowest levels of expression in the MGD group, while the remaining proteins had the lowest expression in the control group. Similarly, in the MGD group, we found overexpression of the following proteins: annexin-A1 (ANXA1), clusterin (CLU), alpha-1-acid glycoprotein 1 (ORM1), and lactoperoxidase (LPO). LPO was the most downregulated protein in the DE group. Finally, this analysis also revealed, both in the DE and MGD groups, reduced expression of several proteins. These were proline-rich protein 1 (PROL1), secretoglobin family 1D member 1 (SCGB1D1), mammaglobin-B (SCGB2A1), lipocalin-1 (LCN1), cystatin-S (CST4), prolactin-inducible protein (PIP), dermcidin (DCD), submaxillary gland androgen-regulated protein 3B (PROL3), galectin-3-binding protein (LGALS3BP), extracellular glycoprotein lacritin (LACRT), and proline-rich protein 4 (PRR4). The lowest levels of expression of these proteins were found in the DE group. For each protein, Table 3 lists fold-changes and the groups with its highest and lowest expression values.

**Figure 2:**
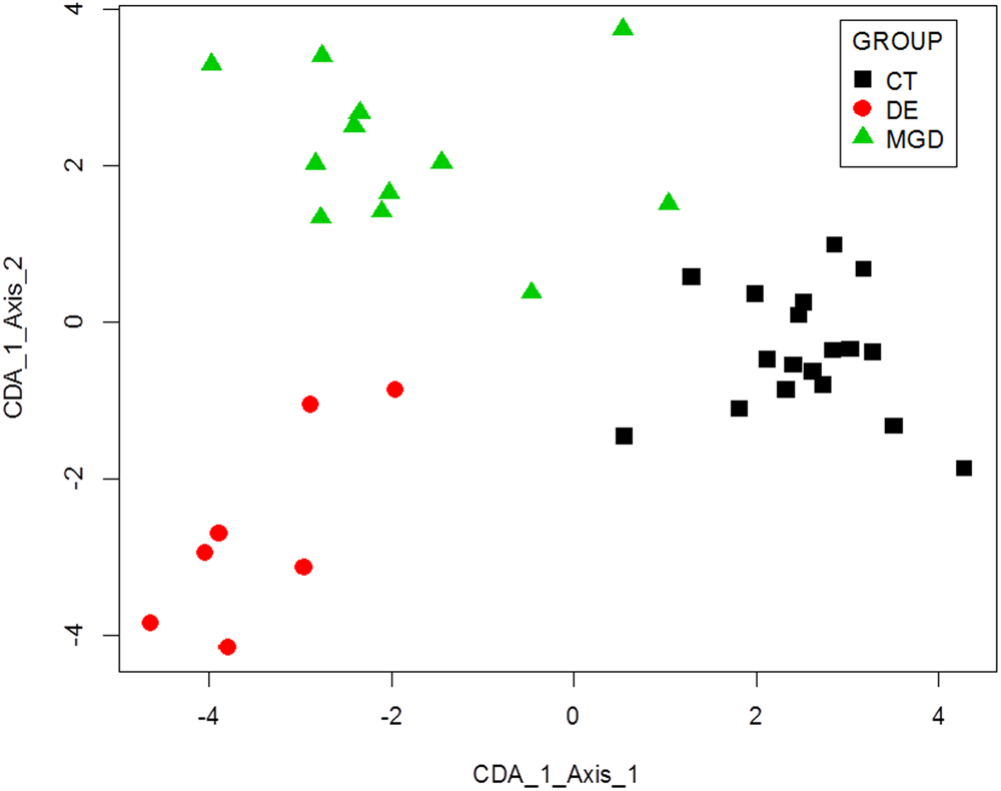
Canonical Discriminant Analysis results showing the separation between the samples as a function of the values for the 26 discriminant proteins obtained by stepwise discriminant analysis. Each of the points represents a sample from each group. Good separation between the groups is apparent. The MGD group was closer to the control group (CT) than the DE group. Key: squares, CT group; triangles, MGD group; circles, DE group.

Using data mining techniques, we also evaluated the spatial separation that these proteins are capable of producing in the three tested groups. We used the expression data of the 26 proteins in the canonical discriminant analysis. We found a good separation between the experimental groups, reflecting the discriminative power presented by this list of proteins. However, it should be noted that there was a larger spatial separation between the DE and control group than between MGD and control (Fig. 2).

### Functional interaction networks

**Figure 3:**
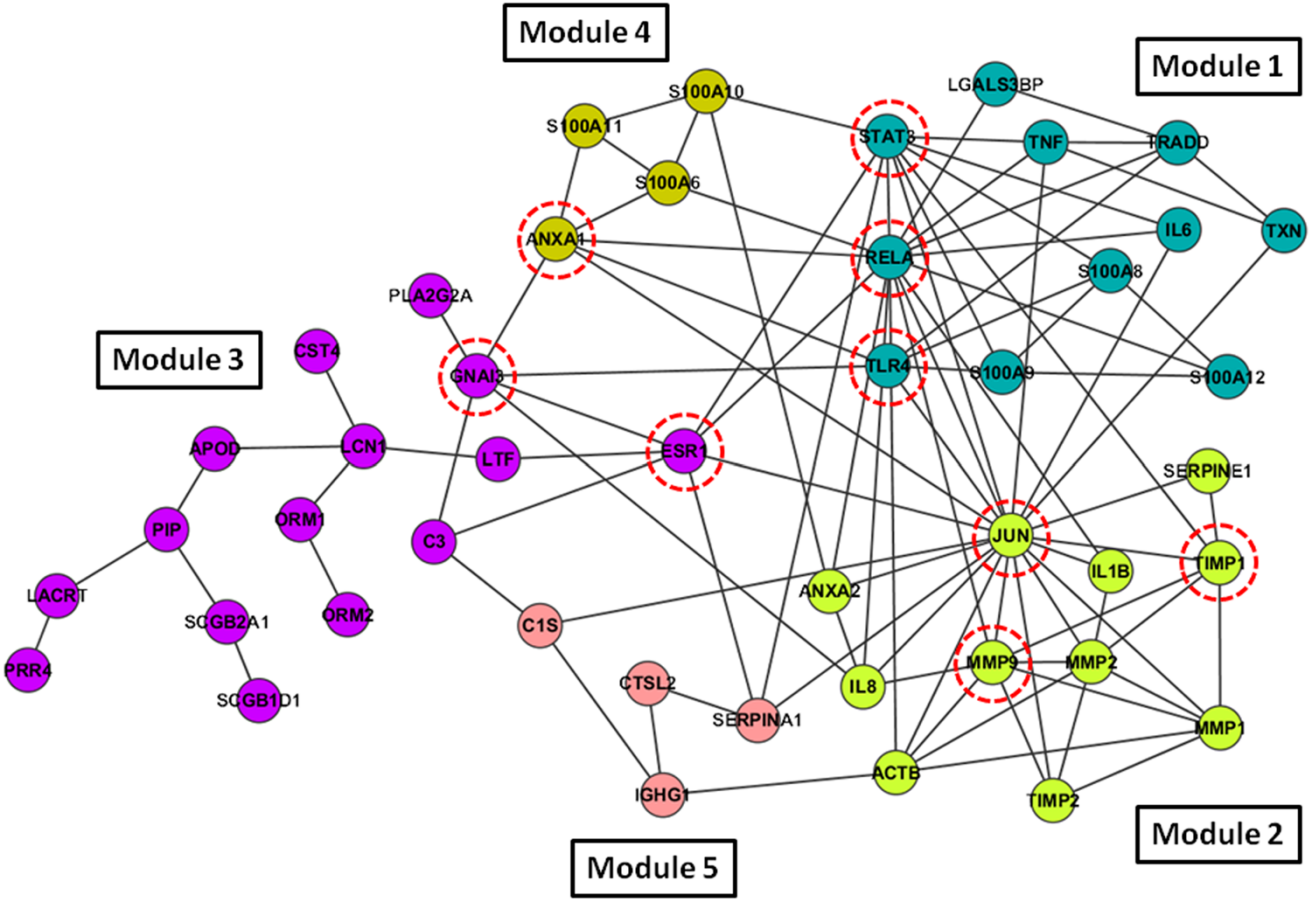
The functional interaction network obtained using the Functional Interaction Reactome Cytoscape plugin^41^. Proteins in the interaction network are represented as nodes (red dashed-line circles), while the interaction between any two proteins is represented by a line. These interactions can be direct (physical) and/or indirect (functional) in nature. Node colors indicate different modules obtained by topological clustering of the network using the functional interaction clustering method.

The Cytoscape Reactome Functional Interaction Network plugin was used to find network patterns by accessing the Reactome Functional Interaction Network database. The disease-related network consisted of 19 of the candidate proteins associated with the diseases, found in this study, and the minimum number of interacting proteins necessary to interconnect them in a network (Fig. 3). Once the network had been constructed, functional clustering was performed using the same Cytoscape plugin. Cluster construction was based on proximity between the nodes. Five topologically well-differentiated modules were obtained (modules 1 to 5), comprising 11, 11, 15, 4, and 4 proteins, respectively. Functional enrichment analyses were then performed for each module to determine the biological processes involved in the pathologies, in relation to the structure of the network. With a false discovery rate cutoff of 0.01, we found statistically significant biological processes in 4 of the 5 modules. As illustrated in Table 4, module 1 of the network mainly represents the processes such as inflammatory response, regulation of chemokine production, response to lipopolysaccharide, cytokine-mediated signaling pathway, apoptotic signaling, acute-phase response, cell redox homeostasis, innate and humoral immune response, regulation of JNK cascade, and cellular defense response. Module 2 was principally formed by proteins involved in the extracellular matrix organization, collagen catabolic process, response to cytokine stimulus, angiogenesis, aging, response to hypoxia, regulation of cell proliferation, and neutrophil chemotaxis. Module 3 was made up of proteins significantly related to the defense response to bacteria and positive regulation of the inflammatory response. Module 4 was related to signal transduction, regulation of apoptosis, and fibroblast proliferation processes. Module 5 did not show significant relationships with any biological processes.

**Table 4:** Involvement of each module in different biological processes, showing the proteins implicated in each process^42^.

Module #	Biological process	# proteins from Module	FDR	Nodes
1	inflammatory response	6	<1.000E-03	IL6, TNF, S100A8, RELA, S100A9, S100A12
	positive regulation of chemokine production	3	<3.333E-04	IL6, TNF, TLR4
	response to lipopolysaccharide	4	2.50E-04	IL6, S100A8, S100A9, TLR4
	cytokine-mediated signaling pathway	3	1.06E-03	IL6, RELA, STAT3
	extrinsic apoptotic signaling pathway	2	9.44E-04	TNF, TRADD
	acute-phase response	2	4.03E-03	IL6, STAT3
	cell redox homeostasis	2	4.26E-03	IL6, TXN
	innate and humoral immune response	4	5.80E-03	RELA, TXN, TLR4, S100A12
	positive regulation of JNK cascade	2	4.20E-02	TNF, TLR4
	cellular defense response	2	4.73E-02	LGALS3BP, RELA
2	extracellular matrix organization	5	<5.000E-04	MMP9, TIMP2, MMP2, MMP1, TIMP1
	collagen catabolic process	3	3.33E-04	MMP9, MMP2, MMP1
	response to cytokine stimulus	3	3.00E-03	JUN, SERPINE1, TIMP1
	angiogenesis	4	2.67E-03	IL8, JUN, MMP2, ANXA2
	aging	3	1.26E-02	JUN, IL1B, TIMP1
	response to hypoxia	2	1.30E-02	MMP9, SERPINE1
	negative regulation of cell proliferation	4	1.22E-02	IL8, JUN, IL1B, TIMP2
	neutrophil chemotaxis	2	1.63E-02	IL8, IL1B
	cellular response to interleukin-1	2	1.49E-02	IL8, MMP9
	response to hypoxia	3	1.72E-02	MMP9, IL1B, MMP2
3	defense response to bacterium	4	3.52E-03	PLA2G2A, CST4, LCN1, LTF
	positive regulation of inflammatory response	5	4.73E-03	C3, APOD, ORM1, ORM2, PLA2GSA
4	signal transduction	4	1.90E-03	S100A6, S100A11, ANXA1, S100A10
	apoptosis	4	1.82E-03	S100A6, S100A11, ANXA1, S100A10
	fibroblast proliferation	4	3.74E-03	S100A6, S100A11, ANXA1, S100A10

FDR = False discovery rate.

Finally, to find the most significant nodes in the network, the BisoGenet plugin was applied to the network. This plugin generates numerical and graphical output, making it easy to find the key nodes in the complex networks. This analysis can provide biologically meaningful node identification and functional classification by identifying the major nodes, the so-called “hub nodes,” which have many incoming and/or outgoing connections within a network. As a result, we found that the proteins MMP9, JUN, RELA, STAT3, TLR4, ESR1, GNAI3, ANXA1, and TIMP1 were the hub nodes of the functional interaction network. These proteins are involved in inflammation, adherens junction process, signaling pathways, cell–cell communication, and signal transduction.

### Validation of candidate biomarkers using microarray assays

**Figure 4:**
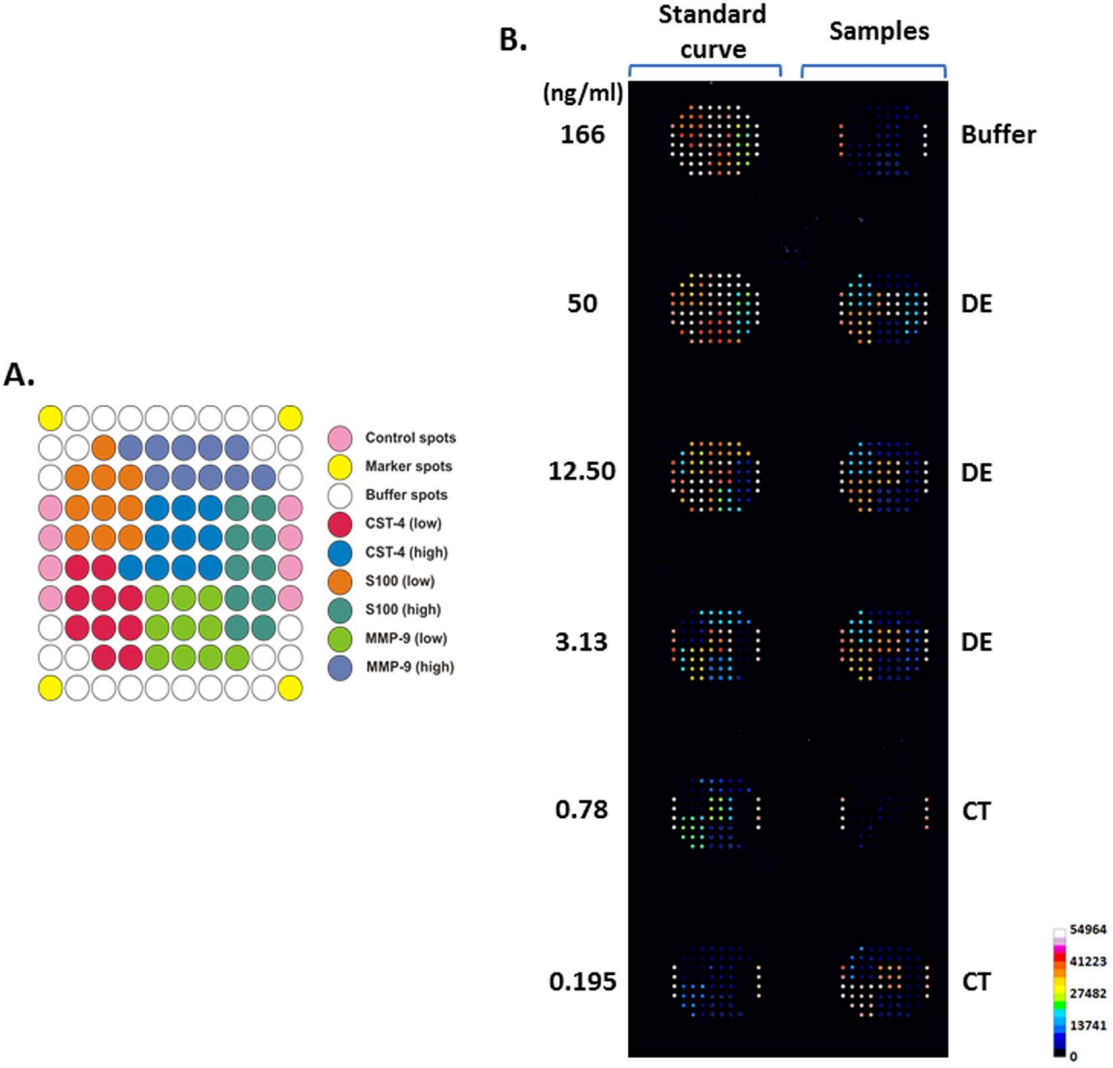
Antibody microarrays. (**A**) Spotting pattern for the 12-subarray format. (**B**) Representative image of the arrays showing the distribution of the standard calibration curve and samples. Each microarray slide contains a standard calibration curve (left column) and the fluorescence acquisition for 5 tear samples. Fluorescence scans of the microarray multiplex assays were acquired at 633 nm.

**Figure 5:**
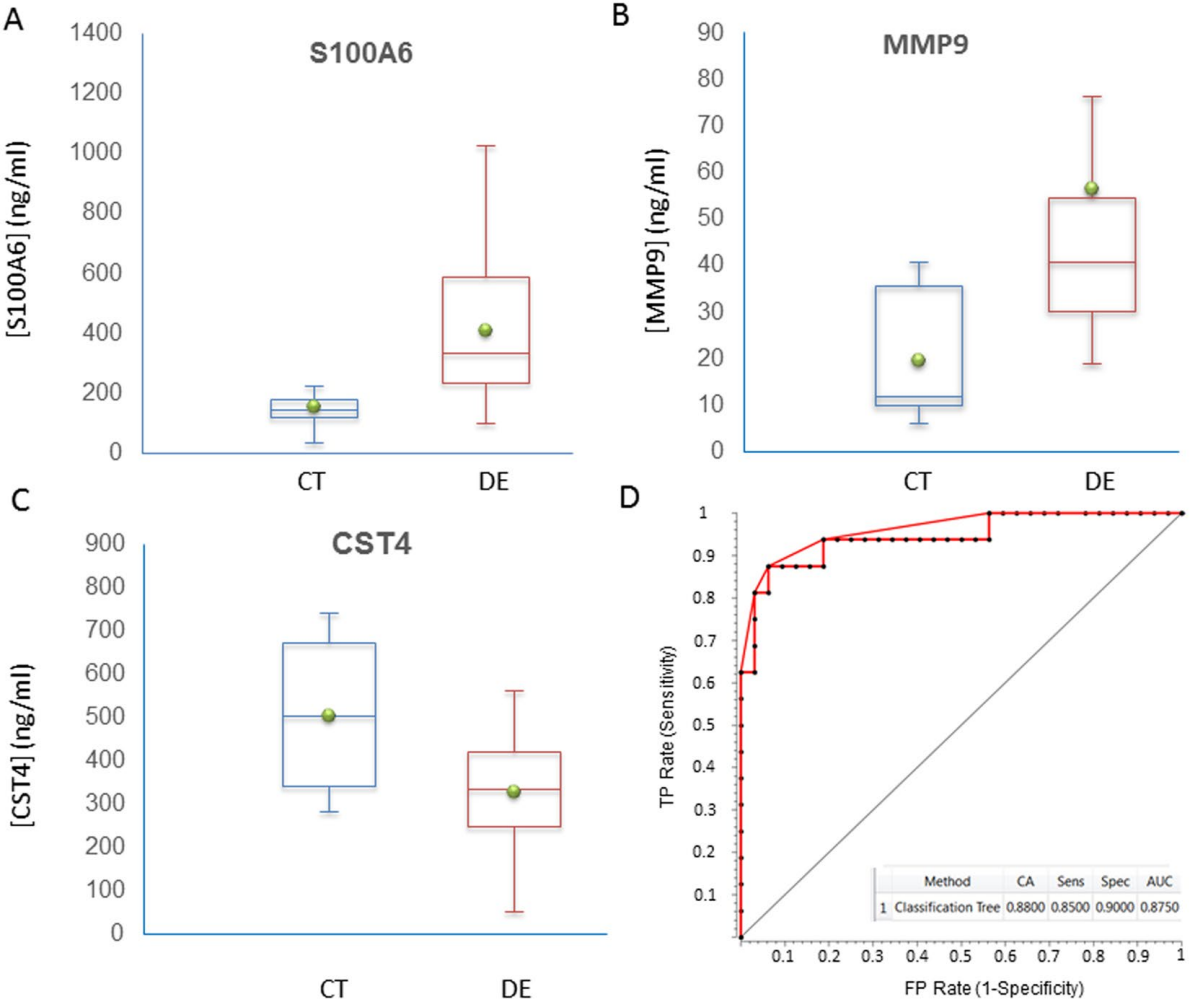
Comparison of the concentrations of candidate biomarkers in control (CT) and dry eye (DE) groups, measured using the customized antibody microarrays. Concentration is expressed in ng/mL. Green circles represent the mean concentration for the group. (**A**) Whisker plot of protein S100A6 concentrations. (**B**) Whisker plot of protein MMP9 concentrations. (**C**) Whisker plot of protein CST4 concentrations. (**D**) ROC curve analysis obtained by logistic regression using the three candidate biomarkers.

To validate further the relative protein expression data of tear proteins biomarkers obtained via MS-based spectral-counting techniques, we employed an orthogonal technique based on antibody microarrays, using an independent group of DE and control samples. The S100A6 and CST4 proteins, significantly deregulated according to the results of the LC-MS/MS analysis, were selected for validation. The MMP9 protein, inferred from network analysis, was also used in this validation. The antibody microarray assays confirmed significant changes in CST4 and S100A6 levels seen in LC-MS/MS assays. They also corroborated the network analysis inference of the implication of MMP9 in the DE disease (Fig. 4). The results showed that the tear concentrations of S100A6 (p-value = 0.0008) and MMP9 (p-value = 0.0097) were significantly higher in the DE group in comparison with the control group, confirming the mass spectrometry findings. However, the CST4 level was significantly lower in the DE group than in the CT group (p-value = 0.0028) (Fig. 5). The ratios between the DE and CT group for S100A6, MMP9, and CST4 were 2.67, 2.48, and −1.59, respectively.

In addition, we used these three markers to generate multivariate predictive models. To this end, we performed training employing random sampling with 70% of the data and a logistic regression algorithm. Subsequently, the classifier method was validated using the remaining 30% of data. The results showed an AUC value of 87.5% (Fig. 5D).

### Correlation of validated tear biomarkers with clinical endpoints

The data obtained for proteins S100A6, CST4, and MMP9 were used to assess the correlation between the biomarkers and clinical parameters. A post-hoc Pearson correlation analysis was performed to determine whether the individual protein levels were associated with the tested clinical parameters (Schirmer I test, TBUT, OSDI, fluorescein staining, and age).

S100A6 and MMP9 showed significant positive correlation with OSDI and fluorescein staining and negative correlation with TBUT and Schirmer I test (Table 5). Similarly, S100A6 and MMP9 biomarkers positively correlated with each other. CST4 protein negatively correlated with fluorescein staining. There was no correlation between the biomarkers and the age of patients.

**Table 5:** Pearson correlation matrix for biomarker concentrations and clinical parameters.

Variables	S100A6	MMP9	CST4	OSDI	TBUT	Schirmer	Fluorescein	Age
S100A6	1							
MMP9	**0.4643**	1						
CST4	−0.1601	0.1108	1					
OSDI	**0.3616**	**0.3861**	−0.0771	1				
TBUT	**−0.7439**	**−0.6303**	0.0431	−0.2816	1			
Schirmer	**−0.5290**	**−0.4459**	0.0800	**−0.6099**	**0.5456**	1		
Fluorescein	**0.5108**	**0.5983**	**−0.3481**	**0.4303**	**−0.5310**	**−0.5179**	1	
Age	0.1708	0.2136	−0.1605	0.4733	−0.1092	−0.3065	**0.4478**	1

Significant correlations are indicated in bold (p-value < 0.05).

## Discussion

In our MS-based proteomic study, we first compared tear samples of DE and MGD patients with control tear samples. The following proteins were mostly overexpressed in DE: C3, S100A6, S100A8, CP, APOD, ORM2, TXN, IGHG1, PLA2G2A, SERPINA1, and SLPI. These proteins are involved in biological processes related to the defense response, inflammatory process, and response to wounding. Among these proteins, TXN, IGHG1, PLA2G2A, SERPINA1, and SLPI were most downregulated in MGD.

The most representative overexpressed proteins in the MGD group were ANXA1, CLU, ORM1, and LPO; the LPO protein had its lowest expression level in the DE group. These proteins are implicated mainly in apoptosis, oxidative stress, immune response, and keratinocyte differentiation, which represent some of the principal processes involved in ocular surface diseases^45^. A panel of TXN, IGHG1, PLA2G2A, SERPINA1, SLPI, and LPO proteins could unequivocally discriminate between DE and MGD as, according to our observations, their regulatory trends in these disease groups are opposite.

TXN is implicated in immune response, cell proliferation, cell–cell signaling, and oxidation–reduction processes^46^. IGHG1 is associated with immune response^47^, and PLA2G2A is involved in the defense and inflammatory responses and lipid catabolic processes^48^. SERPINA1 is involved in acute-phase response, proteolysis and platelet activation^49^, and SLPI, in the defense response^50^. The increased expression of these proteins in DE and the reduction of their levels in MGD suggest an enhanced activation of defense response, inflammatory, proteolytic, or cell–cell signaling processes triggered by a decrease in tear production.

In contrast, LPO is associated with the response to oxidative stress^51^. The direct relationship between hyperosmolarity and oxidative stress has been described by Zhang *et al*.^52^. Gaffney *et al*.^53^ have predicted substantially higher levels of hyperosmolarity in evaporative DE than in aqueous-deficient DE condition, which might explain the high expression levels of LPO protein in MGD group in comparison with DE. This observation and the spatial separation of MGD and DE groups in the canonical discriminant analysis, seen here suggest a different activation of osmolarity compensatory mechanisms, depending on the typology of dry eye.

In summary, the changes in regulatory processes in which these proteins are involved (such as endopeptidase activity, response to bacteria, acute-phase response, cell–cell signaling, or response to oxidative stress) might be the key biological events which distinguish DE from MGD. Validation of those candidate protein biomarkers in new tear samples will be a target of further studies.

In contrast, the expression of another group of proteins was significantly downregulated in both pathologies: PROL1, SCGB1D1, SCGB2A1, LCN1, CST4, PIP, DCD, PROL3, LGALS3BP, LACRT, and PRR4. Most of these are proteins secreted by the lacrimal and Meibomian glands^54,55^. Taking into account the main functions of these proteins, this result suggests an association of the secretory deficiency of aqueous-deficient DE or obstructive MGD with a decreased antibacterial activity, which could increase a risk of ocular infection. Some of these proteins, such as CST4, LCN1, SCGB2A1, and PIP, have also been downregulated in a previous study reported by our group^24^. Clustering analysis revealed that this protein group, characterized by a reduction in their expression levels, could be used to discriminate between DE and MGD.

We also found a very significant separation between the control and DE groups, with MGD occupying an intermediate position between these two groups (as presented in Fig. 2). Interestingly, a similar behavior was observed by our research group in a recent study^56^, in which we compared the conjunctival epithelium proteomes of DE, MGD, and CT individuals. In that work, we observed, based on a group of 17 proteins, a clear separation between DE and CT groups, with MGD in an intermediate position, closest to the CT group. These independent results from tear and epithelium analysis support the hypothesis that tear proteome alterations accompany the changes in conjunctival epithelium.

Apart from the unclear etiology of the studied pathologies, the heterogeneity of the reported results constitutes another key challenge in proteomic studies of complex diseases. Several factors can affect the results obtained for the tear proteome, including the tear collection method and sample storage^29^, analytical methods^30^, and differences in the patient inclusion and exclusion criteria. To validate a robust biomarker, it is of paramount importance to establish its reproducibility using several different technological approaches. To achieve this, we compared our results with those obtained in other proteomic tear studies. Tear samples used in these studies, representing the same pathologies, have been analyzed using 2D-PAGE^24,28,57^, SELDI-TOF profiling^20^, MS-based quantification approaches^7–9,14,17,19,23,25,26,28,58–60^, or ELISA assays^61–63^. The comparison revealed discrepancies between the results of different studies, probably caused by some of the previously mentioned factors. However, the proteins whose expression behavior is similar in different studies acquire greater weight in terms of their robustness as biomarkers. Thus, despite the differences in the analytical technologies employed, we found a group of proteins whose expression was consistently upregulated in the DE group, in comparison with the controls, in the cited studies (S100A8, S100A6, C3, CP, ORM2, IGHG1, SERPINA1, ANXA1, CLU, and ORM1^7–9,14,20,23,24,26,28,58–63^). Similarly, we found another group of proteins with reduced expression levels in the pathological groups, including PIP, proline-rich family (PROL1, PROL3, PRR4), SCGB1D1, CST4, LACRT, and LCN1^7–9,14,17,20,24–26,28,57,60^. The proteins whose expression behavior is consistent in different studies are listed in Table 6.

**Table 6:** Proteins with expression profiles similar to those in other studies.

Expression change	Gene name
Upregulated	S100A8^7,9,14,20,23–26,28,53^
	S100A6^24,25,52^
	C3^8,14,25^
	CP^54–56^
	ORM2 ^14^
	IGHG1 ^8,14^
	SERPINA1^14,20^
	ANXA1^14,23,24,51^
	CLU^25,51^
	ORM1^7,9,14,25^
Downregulated	PIP^7–9,24–26,49^
	PROL1 ^8,14^
	PROL3 ^20^
	PRR4 ^8,14,17,20,23,25,26,53^
	SCGB1D1 ^8,14,17,26,28^
	CST4 ^8,14,17,24,25,49^
	LACRT^8,14,25,26^
	LCN1 ^7–9,17,24,25,28^

The proteins S100A6, ANXA1, and CST4 are particularly noteworthy as molecular markers. Apart from their consistent expression behavior in various studies, their validity has been already confirmed using absolute quantification in ELISA assays with independent samples^24^. That validation study has been conducted using 44 individuals for a biomarker discovery pipeline and 100 for validation. The study has shown a correct assignment of 97.9% in sample classification for a panel of five markers (S100A6, ANXA1, CST4, PLAA, and ANXA11), with a precision of 93.5% for the first three markers. These results confirm the validity of these biomarkers.

Our study also found novel markers associated with ocular surface disorders: APOD, TXN, PLA2G2A, SLPI, LPO, LGALS3BP, and DCD.

Label-free approaches are increasingly considered advantageous and reliable since no additional chemical manipulation is needed. These proteomic methods have gained popularity due to their compatibility with high-throughput systems, their speed, and good reproducibility in the complex peptide mixture analyses^6^. Spectral-counting-based quantification is more reproducible and has a larger dynamic range than other label-free quantification methods^64^. However, in spectral-counting proteomics, physicochemical properties of the peptides can affect MS detection. This might result in imprecise results due to errors in the number of repeat observations of MS/MS spectra of the peptides. Therefore, we applied a peptide-specific normalization factor, which depends on peptide detection probability and improves the accuracy and reproducibility of the analysis^65,66^.

Complex diseases constitute intricate systems of altered biological processes. The study of systems biology using functional interaction networks reveals the changes triggered by various stressors (evaporation, osmolarity, hyposecretion) and the molecular machinery that produces the diseases. Clusters of highly interconnected nodes can be seen within the functional interaction networks; these are often the protein complexes involved in pathways with a high probability of being regulated by the same mechanisms^67^. Our analysis of the functional network revealed five modules implicated in biological processes such as defense, inflammatory and acute-phase responses, response to wounding, response to lipopolysaccharide, T cell secretory granule organization, apoptosis, oxidative stress, extracellular matrix organization, signal transduction, keratinocyte differentiation, and fibroblast proliferation. Similar results of DE tear proteomics analyses have been reported in other studies^24,31,45^. In addition, we identified the most essential nodes as a function of their topological characteristics within the network and their interconnectivity with other nodes^68^. These essential central nodes in the network may provide a detailed insight into the functions involved and their relationships. Our results indicate that DE and MGD share a functional interaction network in which the proteins JUN, RELA, STAT3, TLR4, ESR1, MMP9, GNAI3, ANXA1, and TIMP1 behave as principal nodes. These proteins are implicated in biological processes such as adherens junction processes, Wnt signaling pathways, cell–cell communication, and signal transduction. These processes appear to be associated with the very nature of the studied pathologies^45^.

Using a second, independent analytical method, we confirmed the results of the mass spectrometry analysis for two proteins (S100A6 and CST4). We also included a third potential biomarker inferred from the network analysis (MMP9), a principal node, which has often been reported as related to DE disease^69–73^.

To select the candidate biomarkers to validate in our study, we considered statistical significance (FDR), upregulation and downregulation fold-change, biomarkers representing different physiological processes (biological significance), number of replications (number of studies detecting a significant change), and the value of the protein in the previously reported panels of biomarkers or clinical trials. The S100A6 and CST4 proteins selected in our MS/MS study comply with the established criteria. One of these biomarkers was upregulated (S100A6), and one, downregulated (CST4). They are involved in different physiological processes (S100A6 participates in calcium binding/epithelial integrity and growth and CST4, in cysteine protease inhibitor/antimicrobial activity).

S100A6 has the most significant FDR. Although this protein showed only an intermediate fold-change value in this study, in our previous report using 2D-PAGE^24^, its fold-change was high (8.5). It has a suitable number of replicates (number of studies reporting similar changes): three in unbiased mass spectrometry screenings (US) and one validation in candidate immuno-detection study (CA)^74^. This protein has been included in a panel of DE biomarkers^24^. In addition, S100A6 has been used for monitoring the response of patients to changing glaucoma treatment from preserved latanoprost to preservative-free tafluprost. The increase in the level of tear S100A6 in patients treated with preserved latanoprost was reduced a year after switching to preservative-free tafluprost^75^, suggesting the potential application of this protein not only as a diagnostic but also as a prognostic biomarker (as in cases of topical drug-induced DE)^76^. In this study, CST4 showed a significant (but intermediate-level) FDR and fold-change. However, in our previous analysis using 2D-PAGE, we have found a 4.6-fold change for this protein^24^. In addition, CST4 has a considerable number of replicates: five in unbiased mass spectrometry screenings (US) and one validation in a candidate immuno-detection (CA)^74^. It has been included in a panel of DE biomarkers^24^. Finally, the two biomarkers, when used together as panel, have shown correct assignment (CA) of 86% in the diagnosis and classification of patients (unpublished data).

The MMP9 protein has been found in candidate immuno-detection and activity detection (CA) studies but not in unbiased mass spectrometry screening^74^. We selected this biomarker in our network analysis because of the nine proteins identified as central nodes, MMP9 was the protein best represented in modules (Table 4). Accordingly, the top proteins in the central nodes, in decreasing order of interaction networks (5, 4, 3, and 2), were: MMP9 (module 2) in 5 biological processes (BP); RELA and TRL4 (both in module 1) in 4 BP; ANXA 1 (module 4) and JUN and TIMP1 (module 2) in 3 BP; and STAT 3 (module 1) in 2 BP. Moreover, in module 2, MMP9 was the main player in the nodes with the most significant FDR.

The microarrays were specifically designed for 1-microliter tear sample analysis. The standardization of the miniaturized immunoassays had included several important steps. We selected the high-affinity compatible antibody pairs (using SPR and sandwich ELISA assays) and chose appropriate slide surfaces for the linkage of capture antibodies. We experimentally determined the incubation times and washing and blocking buffers. We also performed cross-reactivity studies to assure specificity of the assays and adjusted the calibration curves to clinically relevant concentration ranges (unpublished data).

The results of microarray analysis corroborated the proteomic results obtained using mass spectrometry and network analysis inference. They were comparable with the results of a study previously published by our group, in which these biomarkers were examined using commercially available ELISA kits^24^. The ROC curve values in the two studies are similar. Here, the results showed a high degree of correlation between the biomarkers and the clinical data, which is essential when considering a biomarker as surrogate endpoint^77^. However, there was no correlation with the age of patients, indicating that the biomarkers are valid for any age range.

Taking into account the scarcity of tear samples (only a few microliters can be obtained from each patient), the microarray technology is a promising alternative to other proteomic techniques for simultaneous protein/peptide quantification. Another important advantage of this technique is that a large number of proteins can be examined at the same time. Multifactorial pathologies such as DE are complex and involve many proteins and biological processes; a simultaneous analysis of multiple proteins in the same sample can provide a broad overview of the changes in protein expression during disease development.

We are currently evaluating the selected biomarkers (using multiplexed quantification techniques) in new tear samples obtained from different pathologies.

In summary, we performed a study of differential protein expression in DE and MGD disorders, using quantitative proteomics based on label-free MS. We studied the behavior of these two pathologies, from a biological perspective, using functional interaction networks. The analysis revealed nine central nodes, represented by the MMP9, JUN, RELA, STAT3, TLR4, ESR1, GNAI3, ANXA1, and TIMP1 proteins. These essential nodes might determine the biological functions to be studied in the future. Such studies should help to understand the diverse pathologies and accelerate the development of new effective therapeutic agents and strategies. Our comparison with the results obtained using different proteomic technologies corroborated our candidates for stable and robust molecular markers of the studied diseases. Among these, the S100A6 and CST4 proteins are some of the most noteworthy. They are now confirmed by orthogonal validation using ELISA^24^ and the customized microarray assays employed here. Thus, they are validated using two different technologies, in different tear samples and in independent assays. Finally, we explored the correlation of selected biomarkers with clinical parameters, obtaining good correlation levels and confirming the validity of these proteins as tear biomarkers for DE. However, additional coordinated interlaboratory validation should be conducted to test the potential tear biomarkers in different populations.
